# The punctate localization of rat Eag1 K^+^ channels is conferred by the proximal post-CNBHD region

**DOI:** 10.1186/1471-2202-15-23

**Published:** 2014-02-04

**Authors:** Chao-Chin Chuang, Guey-Mei Jow, Huei-Min Lin, Yu-Han Weng, Jui-Hsiang Hu, Yi-Jheng Peng, Yi-Chih Chiu, Mei-Miao Chiu, Chung-Jiuan Jeng

**Affiliations:** 1 Institute of Anatomy and Cell Biology, School of Medicine, National Yang-Ming University, No. 155, Section 2, Li-Non Street, Taipei 12212, Taiwan; 2 Brain Research Center, National Yang-Ming University, No. 155, Section 2, Li-Non Street, Taipei 12212, Taiwan; 3 School of Medicine, Fu-Jen Catholic University, Hsin-Chuang, Taipei County 24205, Taiwan; 4 Current address: Department of Physiology, College of Medicine, National Taiwan University, Taipei, Taiwan

**Keywords:** Hippocampal neuron culture, Subcellular localization, Immunofluorescence, Synaptosomal fractionation, Electrophysiology

## Abstract

**Background:**

In mammals, Eag K^+^ channels (K_V_10) are exclusively expressed in the brain and comprise two isoforms: Eag1 (K_V_10.1) and Eag2 (K_V_10.2). Despite their wide presence in various regions of the brain, the functional role of Eag K^+^ channels remains obscure. Here we address this question by characterizing the subcellular localization of rat Eag1 (rEag1) and rat Eag2 (rEag2) in hippocampal neurons, as well as determining the structural basis underlying their different localization patterns.

**Results:**

Immunofluorescence analysis of young and mature hippocampal neurons in culture revealed that endogenous rEag1 and rEag2 K^+^ channels were present in both the dendrosomatic and the axonal compartments. Only rEag1 channels displayed a punctate immunostaining pattern and showed significant co-localization with PSD-95. Subcellular fractionation analysis further demonstrated a distinct enrichment of rEag1 in the synaptosomal fraction. Over-expression of recombinant GFP-tagged Eag constructs in hippocampal neurons also showed a significant punctate localization of rEag1 channels. To identify the protein region dictating the Eag channel subcellular localization pattern, we generated a variety of different chimeric constructs between rEag1 and rEag2. Quantitative studies of neurons over-expressing these GFP-tagged chimeras indicated that punctate localization was conferred by a segment (A723-R807) within the proximal post-cyclic nucleotide-binding homology domain (post-CNBHD) region in the rEag1 carboxyl terminus.

**Conclusions:**

Our findings suggest that Eag1 and Eag2 K^+^ channels may modulate membrane excitability in both the dendrosomatic and the axonal compartments and that Eag1 may additionally regulate neurotransmitter release and postsynaptic signaling. Furthermore, we present the first evidence showing that the proximal post-CNBHD region seems to govern the Eag K^+^ channel subcellular localization pattern.

## Background

Voltage-gated potassium (K^+^) channels play various essential physiological roles in neurons, including controlling neuronal excitability, setting neuronal firing frequencies, shaping action potential waveforms, and modulating neurotransmitter release [1]. The *ether-à-go-go* (Eag) K^+^ channel belongs to the EAG family of voltage-gated K^+^ channels that comprises three gene subfamilies; these are *eag* (K_V_10), *erg* (*eag*-related gene)(K_V_11), and *elk* (*eag*-like K^+^ channel)(K_V_12) [2]. Results from *in situ* hybridization studies have indicated that Eag is neuron-specific K^+^ channel that is widely expressed in various regions of the brain [3-6]. In mammals, two Eag isoforms have been identified: Eag1 (K_V_10.1) and Eag2 (K_V_10.2) [4,6-9].

Despite their abundant expression in the brain, the functional significance of Eag1 and Eag2 K^+^ channels remains obscure. One strategy to ascertain the neurophysiological role of voltage-gated ion channels is to identify their subcellular localization in neurons [10,11]. Previous immunofluorescence characterization carried out in our laboratory has demonstrated that rat Eag1 (rEag1) and rat Eag2 (rEag2) K^+^ channels have different subcellular localizations over the dendrosomatic compartment in both hippocampal neurons and the retina [12,13]; specifically, rEag1 channels exhibit a much broader range of expression, extending from somas to distal dendrites, and show a distinct punctate localization pattern. Since this punctate staining is co-localized with the presynaptic vesicle protein synaptophysin and the postsynaptic density protein densin-180 [12], it is likely that rEag1 is present within the synaptic region. Given that our previous immunofluorescence study was conducted with 14 days *in vitro* (DIV14) hippocampal cultures, wherein extensive neuronal connections are already formed, it was not possible to determine precisely whether rEag1 and/or rEag2 show axonal localization. A recent report applying immunofluorescence and quantum dot technology to DIV10 hippocampal neurons confirmed the punctate expression and synaptic localization of rEag1 channels [14]; moreover, this study also found that the immunofluorescence staining of rEag1 is co-localized with that of the axon marker tau, raising the possibility that rEag1 channels may be present in axons.

Emerging evidence suggests that specific sequence motifs within channel proteins may govern the subcellular distribution of ion channels in neurons [11,15]. The proteins rEag1 and rEag2 share about 70% identity in amino acid sequence and thus belong to the same EAG K^+^ channel subfamily [4,6]. Nevertheless, it remains unclear what constitutes the structural basis that dictates the differential subcellular localization of these two closely related voltage-gated K^+^ channels. In this study, we addressed this question by generating chimeras between rEag1 and rEag2 K^+^ channels. Quantitative analysis of these chimeras indicates that the proximal post-cyclic nucleotide-binding homology domain (post-CNBHD) region in the carboxyl (C) terminus confers the punctate localization of rEag1 K^+^ channels in hippocampal neurons.

## Methods

### Antibodies

The antibodies used in this study include rabbit anti-rEag1, rabbit anti-rEag2 (Alomone), mouse anti-β-actin (Sigma), mouse anti-MAP2 (Sigma), mouse anti-tau (Chemicon), mouse anti-PSD-95 (Cell Signaling), and mouse anti-synaptophysin (a kind gift from Dr. Erik Schweitzer, UCLA). The specificity of the anti-rEag1 and the anti-rEag2 antibodies has been previously verified [12].

### Animals and hippocampal cultures

All procedures were in accordance with the Guidelines for the Care and Use of Mammals in Neuroscience and Behavioral Research (National Research Council 2003) and approved by the Institutional Animal Care and Use Committee (IACUC) of National Yang-Ming University.

15-day pregnant Sprague–Dawley rats were purchased from the Facility for Animal Research of the National Yang-Ming University. Dissociated hippocampal cultures were prepared using a previously described protocol [16] with a minor modification [12]. In brief, hippocampi were dissected from the brains of embryonic day 18 (E18) embryos, the brains of which were removed and placed in the Hank’s balanced salt solution that contains 10 mM HEPES (pH 7.4) and 1 mM sodium pyruvate. The hippocampus was dissected out and dissociated by incubation with 0.25% trypsin solution. The dissociated cells were plated on coverslips at a density of 200 and 1000 cells/mm^2^ for immunofluorescence and DNA transfection, respectively. Coverslips were coated with poly-D-lysine (1 mg/ml) (Sigma) and laminin (15 μg/ml) (Sigma). Cultures were maintained in the Neurobasal media supplemented with B27 (2%) and glutamax I (0.5 mM) (Invitrogen) in a humidified 5% CO_2_ incubator at 37°C.

Adult female *Xenopus laevis* (African Xenopus Facility) were anesthetized by immersion in ice water containing Tricaine (1.5 g/liter). Ovarian follicles were removed from *Xenopus* frogs, cut into small pieces, and incubated in the ND96 solution [(in mM) 96 NaCl, 2 KCl, 1.8 MgCl_2_, 1.8 CaCl_2_, and 5 HEPES, pH 7.2]. To remove the follicular membrane, *Xenopus* oocytes were incubated in the Ca^2+^-free ND96 solution containing collagenase (2 mg/ml) on an orbital shaker (~200 rpm) for about 60-90 min at room temperature. After several washes with collagenase-free, Ca^2+^-free ND96, oocytes were transferred to ND96. Stage V-VI *Xenopus* oocytes were then selected for cRNA injection.

### Molecular biology

The cDNAs for rEag1 and rEag2 K^+^ channel subunits were kindly provided by Dr. Olaf Pongs (Institute fur Neurale, Signalverarbeitung, Zentrum fur Molekulare Neurobiologie). Green fluorescent protein (GFP)-tagged rEag1 and rEag2 constructs were made by subcloning the full length rEag1 and rEag2 cDNAs into the pEGFP mammalian expression vector (Clontech).

The design of the chimeras between rEag1 and rEag2 were based on sequence alignment. Chimeric channels were constructed by using the overlap PCR mutagenesis method. All constructs were verified by DNA sequencing (Genome Research Center, National Yang-Ming University).

For DNA transfection, human embryonic kidney (HEK) 293 T cells were maintained in DMEM (Invitrogen) supplemented with 2 mM L-glutamine, 100 units/ml penicillin/streptomycin, and 10% (v/v) fetal bovine serum (Hyclone). For immunofluorescence and electrophysiology, cells were grown on poly-lysine-coated coverslips. After 24 hrs, HEK293T cells were transiently transfected with cDNAs by using the Lipofectamine 2000 (LF2000) reagent (Life Technologies).

Cultured hippocampal neurons at 7 days *in vitro* (DIV7) were also transfected by using LF2000. Briefly, various expression constructs were incubated with the LF2000 reagent for 20 min at room temperature. DNA-lipofectamine diluted in the complete medium was added to neuron culture wells. After 4-hr incubation at 37°C under 5% CO_2_, cells were washed gently three times with the culture media and maintained in the incubator before being examined under a fluorescence microscope.

For *in vitro* transcription, cDNAs were linearized with *Not*I. Capped cRNAs were transcribed *in vitro* from the linearized cDNA template with the mMessage mMachine T7 kit (Ambion). The apparent molecular weight and concentration of cRNAs were verified with gel electrophoresis and determined by spectrophotometry, respectively. For cRNA injection, the total volume of injection was always 41.4 nl per *Xenopus* oocyte. Injected oocytes were stored at 16°C in ND96.

### Immunofluorescence

Coverslips containing HEK293T cells or hippocampal neurons were rinsed in PBS [(in mM) 136 NaCl, 2.5 KCl, 1.5 KH_2_PO_4,_ 6.5 Na_2_HPO_4_, pH 7.4] and then fixed with 4% paraformaldehyde in PBS at 4°C for 20 min. Cells were then permeabilized and blocked with a blocking buffer (5% normal goat serum in 20 mM phosphate buffer, pH 7.4, 0.1% (v/v) Triton X-100, and 0.45 M NaCl) for 60 min at 4°C. Appropriate dilutions of primary antibodies were applied in the blocking buffer overnight at 4°C. Immunoreactivities were visualized with goat-anti-mouse antibodies conjugated to Alexa568 or with goat anti-rabbit antibodies conjugated to Alexa488 (Molecular Probes). The fluorescence images were viewed and acquired with a Leica TCS SP5 laser-scanning confocal microscope.

Image analyses were performed with the ImageJ software (National Institute of Health). To determine the number of immunofluorescence clusters per fixed length of neurite, built-in “set scale” and “freehand tool” functions of the software were applied to trace multiple 100-μm neurite segments, followed by counting the number of PSD-95/rEag1/rEag2 puncta within each 100-μm neurite segment. Co-localization of PSD-95 (appearing as red punctate pixels) and rEag1/rEag2 (appearing as green punctate pixels) puncta within each 100-μm neurite segment was recognized by identifying the presence of overlapping punctate pixels. For neurons transfected with various GFP-tagged constructs, the number of GFP puncta per neuron was also estimated using ImageJ. Statistical analyses were executed with the Origin 7.0 software (Microcal Software). All numerical data are shown as mean ± standard error (SEM).

### Subcellular fractionation of rat brain and preparation of PSDs

Subcellular and PSD fractions of adult rat brains were prepared as described previously [17]. In brief, adult rat forebrains were homogenized in the buffer H1 [(in mM) 320 sucrose, 1 NaHCO_3_, 0.5 CaCl_2_, 0.1 PMSF] containing a cocktail of protease inhibitors (Roche) and centrifuged at 1,400×g to remove nuclei and other large debris (P1). The S1 fraction was subject to centrifugation at 13,800xg to obtain the crude synaptosome fraction (P2). The pellet was resuspended in the buffer H2 [(in mM) 0.32 M sucrose and 1 mM NaHCO_3_)] and layered onto the top of the discontinuous sucrose density gradient by using 0.85, 1.0, and 1.2 M sucrose layers. The gradient was centrifuged at 65,000xg for 2 hrs in a Beckman Instruments SW-28 rotor and the synaptosomal fraction (SPM) was recovered from the 1.0-1.2 M sucrose interface. The synaptosomal fraction was extracted in ice-cold 0.5% Triton X-100/50 mM Tris–HCl (pH 7.9) for 15 min and centrifuged at 32,000xg for 45 min to obtain the PSD I pellet. The pellet was resuspended and further extracted a second time with 0.5% Triton X-100/50 mM Tris–HCl (pH 7.9), followed by centrifugation at 200,000×g for 45 min to obtain the PSD II pellet. Protein concentration was determined by the BCA protein assay kit (Thermo). For immunoblotting, 25 μg (H, S1, P2, and SPM) or 5 μg (SPM, PSD I, and PSD II) of proteins were separated by SDS-PAGE, blotted onto nitrocellulose membranes, incubated with the primary antibodies, and imaged with the enhanced chemiluminescence method (Thermo).

### Electrophysiology

For HEK293T cells, conventional whole-cell patch clamp technique was used to record Eag K^+^ currents as described previously [18]. In brief, recordings were performed at 24-48 hrs post-transfection. Patch electrodes with a resistance of ~4 MΩ were pulled on a Narishige PP-830 electrode puller and were filled with a solution containing (in mM) 140 KCl, 1 MgCl2, 10 EGTA, 10 HEPES, pH 7.2. External bath solution comprised (in mM) 140 NaCl, 5 KCl, 1 CaCl_2_, and 10 HEPES, pH 7.2.

Conventional two-electrode voltage clamp recording in *Xenopus* oocytes were performed as described previously [18]. In brief, 2-3 days after cRNA injection, oocytes were functionally assayed in a recording bath containing about ~200 μl of the Ringer solution [(in mM): 115 NaCl, 3 KCl, 1.8 CaCl_2_, 10 HEPES, pH 7.2]. An agarose bridge was used to connect the bath solution with a ground chamber (containing 3 M KCl) into which two ground electrodes were inserted. Borosilicate electrodes (0.1 –1 MΩ) used in voltage recording and current injection were filled with 3 M KCl.

Voltage-clamp protocols were applied with the pCLAMP 8.2/9.0 software (Molecular Devices). Data were acquired with an Axopatch 200A amplifier (Molecular Devices) (for HEK293T cells) or OC-725C oocyte clamp (Warner) (for *Xenopus* oocytes), followed by digitization at 10 kHz with the Digidata 1320A/1322A system (Molecular Devices). Also by using the pCLAMP 8.2/9.0 software, data were filtered at 1 kHz and passive membrane properties were compensated with the -P/4 leak subtraction method. All recordings were performed at room temperature (20-22°C).

Cells with large currents in which voltage clamp errors might appear were excluded from data analyses. Kinetic fitting of Eag K^+^ current traces were implemented with the pCLAMP 8.2/9.0 software. Subsequent numerical analyses and data plotting were performed with the Origin 7.0 software. All numerical data are shown as mean ± SEM.

## Results

### Different localizations of rEag1 and rEag2 channels in axons and synapses

**Figure 1:**
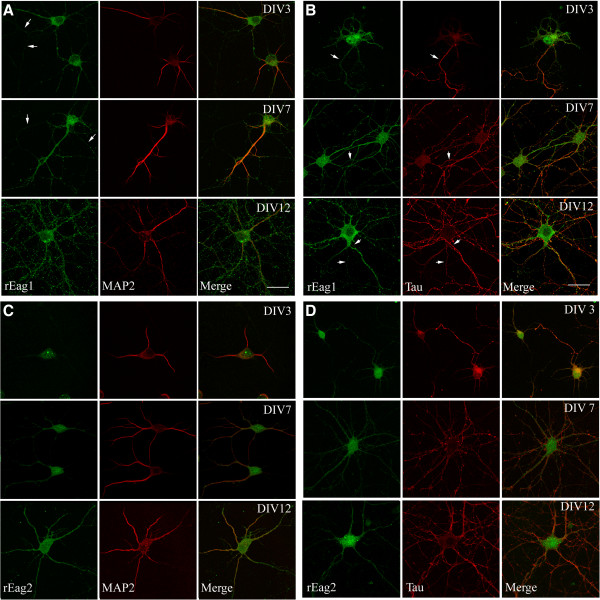
**Subcellular localization of native rEag1 and rEag2 channels in young and mature hippocampal neurons in culture.** Dissociated hippocampal neurons at DIV3, DIV7, and DIV12 were immunostained with the anti-rEag1 **(A, B)** or the anti-rEag2 **(C, D)** antibodies (*shown in green; left panels*), followed by counterstaining with the antibody for the dendritic marker MAP2 or the axonal marker tau (*shown in red; middle panels*). Merged images are shown in the right panels. **(A)** rEag1 immunoreactivities were localized in cell bodies, as well as in MAP2-positive and MAP2-negative (*arrows*) processes. **(B)** rEag1 immunoreactivities were present in the axonal compartment that was clearly defined by the immunofluorescence signal of tau (*arrows*). For DIV12 neurons in both (A) and (B), note the presence of punctate rEag1 staining patterns throughout proximal and distal neurites. **(C, D)** rEag2 immunoreactivities were present in both MAP2-positive and tau-positive processes. No significant rEag2 puncta were observed. Scale bar, 25 μm.

We began by asking whether rEag1 and rEag2 K^+^ channels are present in axons. For isolated hippocampal neurons, the process of axon-dendrite polarization initiates within the first 36 hours in culture and by 72 hours in culture, a single neurite undergoing fast elongation has become an axon [19]. We therefore decided to perform immunofluorescence characterization in DIV3, DIV7, and DIV12 neurons. As illustrated in Figure 1A, rEag1 staining was found to co-localize with the dendrite marker microtubule-associated protein 2 (MAP2) in all three populations of cultured hippocampal neurons, which is consistent with our previous observation that rEag1 K^+^ channels are present in the dendrosomatic compartment. Furthermore, the characteristic punctate localization of rEag1 channels was visible in DIV7 neurons and was profusely present in DIV12 neurons. Interestingly, by closely inspecting the DIV3 and DIV7 neurons, whose neurite networks were less sophisticated, we also observed significant rEag1 immunofluorescence signal in MAP2-negative neurites, which implies that rEag1 channels may be present in axons as well. We then compared the immunofluorescence signals of rEag1 with those of the axon marker tau. Figure 1B shows that in DIV3 neurons, where the location of the axon is clearly defined by the tau immunofluorescence signal, rEag1 channels are present within the soma and also in the complete axonal compartment. A similar tau-positive rEag1 immunostaining pattern can be observed in DIV7 and DIV12 neurons as well, although a significant fraction of the immunofluorescence signal may represent the presence of the channel protein in axons stemming from neighboring neurons. These findings thus demonstrate that rEag1 channels are universally distributed in both the dendrosomatic and the axonal compartments of hippocampal neurons.

We also found that rEag2 was co-localized with MAP2 in all three populations of cultured hippocampal neurons (Figure 1C). In addition, we found that a fraction of the immunofluorescence signal of rEag2 was co-localized with that of the tau immunofluorescence signal, especially in immature DIV3 neurons (Figure 1D). In contrast to rEag1, which was found to be universally present throughout the axonal compartment, in DIV7 and DIV12 neurons, rEag2 seemed to display a pattern in axons that was relatively restricted and showed a low overall level of expression (Figure 1D). Most importantly, in virtually all the neuron samples we analyzed, rEag2 did not show a significant punctate distribution within either the dendrosomatic or the axonal compartments.

**Figure 2:**
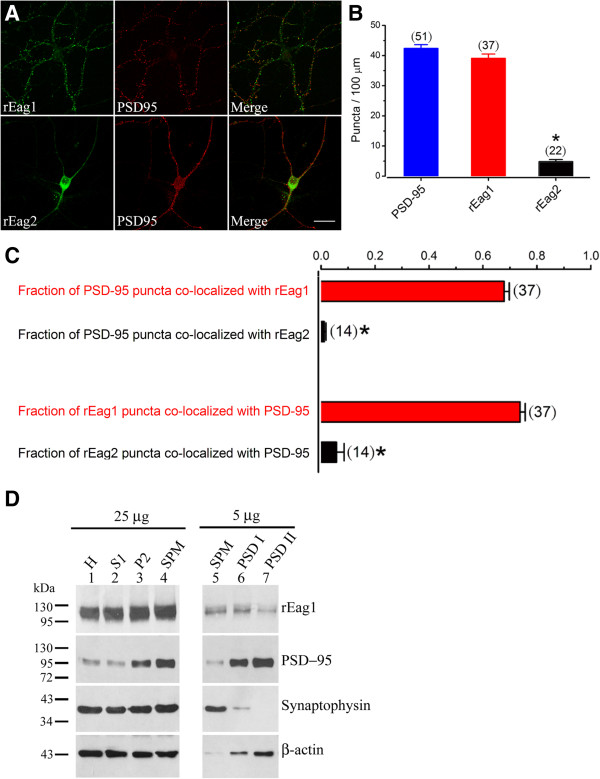
**Synaptic localization of rEag1 channels. (A)** Hippocampal neurons were double-stained for rEag1/rEag2 (*left panels*) and the postsynaptic density marker PSD-95 (*middle panels*). Scale bar, 25 μm. **(B)** Quantification of the number of puncta per 100-μm neurite (puncta/100 μm) for PSD-95, rEag1, and rEag2. The number in parenthesis denotes the amount of neurites analyzed, and the asterisk indicates a significant difference (*t*-test, p < 0.05) from PSD-95. Data were collected from 7-11 different neurons. **(C)** Quantification of the co-localization of PSD-95 with rEag1 or rEag2. The data illustrate the fraction of PSD-95 puncta that were co-localized with rEag1/2 puncta, as well as the fraction of rEag1/2 puncta that were co-localized with PSD-95 puncta. The number in parenthesis denotes the amount of neurites analyzed, and the asterisk indicates a significant difference (*t*-test, p < 0.05) from rEag1. Data were collected from 7-8 different neurons. **(D)** Subcellular fractionation of rat brains: the homogenate (H), the soluble fraction (S1), the crude membrane fraction (P2), the synaptosomal fraction (SPM), and the two postsynaptic density (PSD) preparations (PSD I: one Triton X-100 wash; PSD II: two Triton X-100 washes). The left panel (*25 μg*) illustrates the primary fractionation profile, whereas the right panel (*5 μg*) exemplifies the further enrichment pattern in the three sub-fractions of synaptosomes. All fractions were subject to immunoblotting analyses with the indicated antibodies. 25 μg and 5 μg refer to the amount of total protein loaded in each lane.

As mentioned above, the punctate localization of rEag1 channels was copious in DIV12 neurons, which are known to form numerous and widespread synaptic connections. We quantified the rEag1 puncta within the neurons by calculating the puncta density, which was defined as the number of immunofluorescence puncta per 100-μm neurite (see Methods for detail). The puncta density of rEag1 was about 39 ± 1 (mean ± SEM), which is quite similar to that of the postsynaptic density (PSD) marker PSD-95 (about 42 ± 1) (Figure 2A-B). In contrast, the puncta density of rEag2 was only about 5 ± 1 (Figure 2B). This lack of punctate staining pattern seems to imply that rEag2 is not significantly present at synapses. Consistent with this notion, only about 1 ± 1% of PSD-95 puncta were found to be co-localized with rEag2 puncta, and conversely the PSD-95 co-localization ratio of rEag2 puncta was only about 6 ± 3% (Figure 2C). This contrasts with about 68 ± 2% of PSD-95 puncta being co-localized with rEag1 puncta, and with about 74 ± 2% of rEag1 puncta being co-localized with PSD-95 puncta (Figure 2C).

An alternative approach to addressing the synaptic localization of proteins is to examine their subcellular fractionation. By this approach, the differential localization of synapse-related proteins can be demonstrated via their distinct enrichment patterns in the synaptosomal (SPM) and the two PSD (PSD I and PSD II) fractions. As depicted in Figure 2D, the presynaptic marker synaptophysin was highly enriched in the SPM fraction and was virtually absent in the PSD II fraction. In contrast, PSD-95, as well as β-actin, was found to be highly enriched in both the PSD I and the PSD II fractions. Moreover, Figure 2D clearly demonstrates that rEag1 protein was significantly enriched in all three of the synaptosomal sub-fractions. Taken together, these findings imply that a significant number of rEag1 K^+^ channels may be present at presynaptic axonal terminals and/or on the postsynaptic dendritic spines.

### Differential subcellular localization of GFP-tagged rEag1 and rEag2 channels in hippocampal neurons

**Figure 3:**
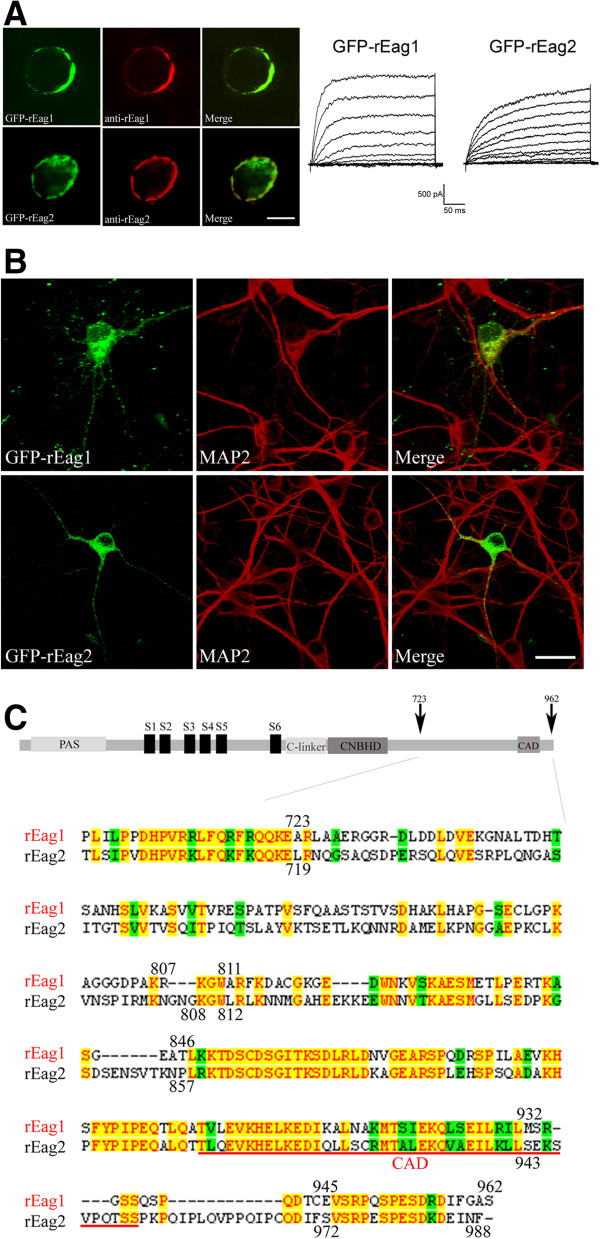
**Expression of GFP-rEag1 and GFP-rEag2 channels in HEK293T cells and hippocampal neurons. (A)** Immunofluorescence staining and functional expression of GFP-rEag1 and GFP-rEag2 K^+^ channels in HEK293T cells. GFP fluorescence (*shown in green*) and rEag1/rEag2 immunofluorescence (*shown in red*) signals demonstrated lucid co-localization at the membrane region. Scale bar, 10 μm. Whole-cell patch clamp parameters: the holding potential for rEag1 and rEag2 was -90 and -110 mV, respectively; the pulse protocol comprised 300-ms depolarizing test pulses ranging from -70 to +50 mV (rEag1) or from -90 to +30 mV (rEag2), with 10-mV increments. **(B)** Over-expression of GFP-rEag1/rEag2 in DIV12 hippocampal neurons. GFP signal is shown in green, and MAP2 immunofluorescence signal in red. Note the presence of prominent GFP puncta for rEag1, but not rEag2. Scale bar, 25 μm. **(C)** (*Top*) Schematic representation of the structural topology of Eag K^+^ channel. (*Bottom*) Protein sequence alignment between rEag1 and rEag2 over the post-CNBHD region. Yellow shade: identical residues. Green shade: homologous residues. Sequence alignment analysis was implemented with the Vector NTI software (InforMax).

To further demonstrate that the punctate localization pattern was indeed an innate distinction between the two channel isoforms, we studied next the exogenous over-expression of rEag1 and rEag2 proteins in hippocampal neurons. A GFP-tag was engineered to be attached to the amino (N) terminus of both rEag1 and rEag2. Upon over-expression in HEK293T cells, the GFP-tagged constructs displayed a clear membrane localization pattern and produced functional K^+^ currents (Figure 3A); these findings indicate that the membrane trafficking and biophysical properties of the GFP-tagged channels are similar to those of their wild-type counterparts. The cDNAs encoding the GFP-tagged proteins were then transfected into DIV7 neurons and this was followed by confocal microscopic analyses at 5 days post-transfection. As shown in Figure 3B (see also Additional file 1), over-expressed GFP-rEag1 channels, but not over-expressed GFP-rEag2 channels, displayed significant punctate localization in DIV12 neurons. These findings are reminiscent of the differential subcellular localization of endogenous rEag1 and rEag2 channels.

### Characterization of the subcellular localization of chimeric channels in hippocampal neurons

**Figure 4:**
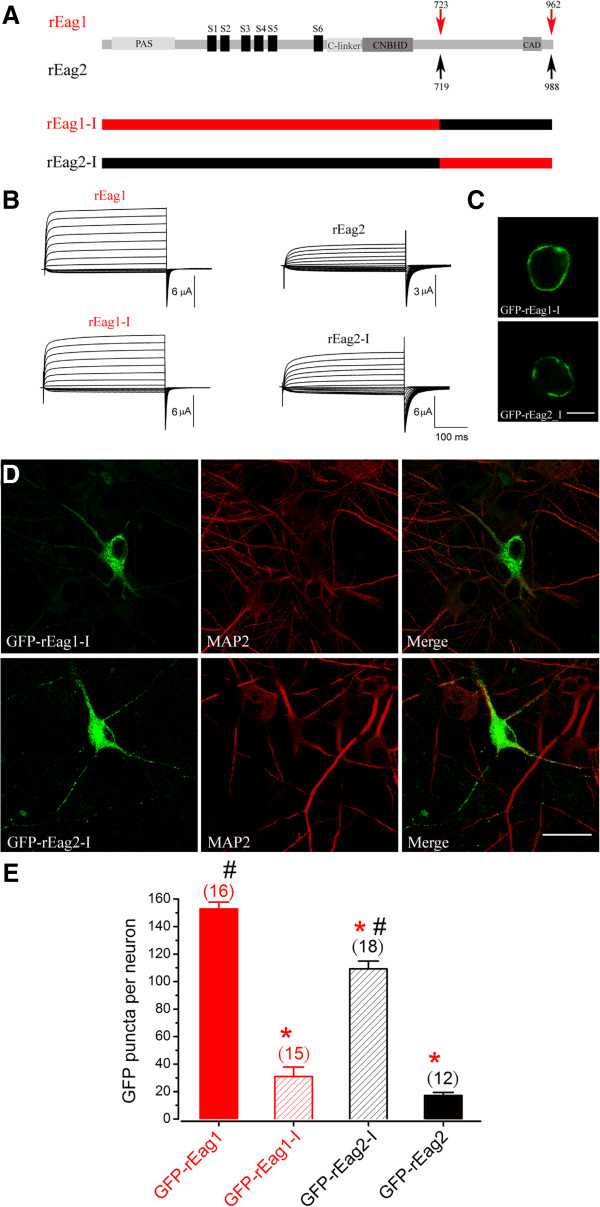
**Characterization of rEag1-I and rEag2-I chimeric channels. (A)** Schematic representation of the construction of rEag1-I and rEag2-I chimeras. For all schematic cartoons hereafter, rEag1 and rEag2 sequences are shown in red and black, respectively. **(B)** Representative K^+^ currents recorded from *Xenopus* oocytes over-expressing the indicated Eag constructs. Two-electrode voltage clamp parameters: the holding potential for rEag1 and rEag2 was -90 and -110 mV, respectively; the pulse protocol comprised 300-ms depolarizing test pulses ranging from -70 to +60 mV (rEag1) or from -100 to +40 mV (rEag2), with 10-mV increments. **(C)** Membrane localization of GFP-rEag1-I/rEag2-I channels in HEK293T cells. Scale bar, 10 μm. **(D)** Expression of GFP-rEag1-I/rEag2-I channels in DIV12 hippocampal neurons. Scale bar, 25 μm. **(E)** Quantification of the number of GFP puncta per neuron for GFP-rEag1, GFP-rEag1-I, GFP-rEag2-I, and GFP-rEag2. The number in parenthesis denotes the amount of neurons analyzed. (*: significantly different from GFP-rEag1; *t*-test, p < 0.05)(#: significantly different from GFP-rEag2; *t*-test, p < 0.05)

Both endogenous and over-expressed rEag1 channels displayed a punctate localization, which strongly suggests that one or more specific sequence motifs within its amino acid sequence may govern the subcellular distribution of the K^+^ channel in neurons. Although rEag1 and rEag2 share about 70% identity in amino acid sequence [4,6], a significant sequence divergence is present within the C-terminal post-CNBHD region, especially from residues A723 through S962 in rEag1 *vs.* L719 through F988 in rEag2 (Figure 3C). To test the hypothesis that a key structural domain within the post-CNBHD region may contribute to the punctate localization of rEag1 K^+^ channels, we generated two chimeric constructs (chimera rEag1-I and chimera rEag2-I) by exchanging the majority of the divergent post-CNBHD sequences between the two channel isoforms (Figure 4A). Upon over-expression in HEK293T cells or *Xenopus* oocytes, both of the chimeric channels yielded significant K^+^ currents and effective membrane surface expression (Figure 4B-C). We then over-expressed these GFP-tagged chimeras in hippocampal neurons in order to study their subcellular localization patterns. Confocal microscopic analysis revealed that the GFP-rEag1-I chimera displayed a rEag2-like pattern with only a few GFP puncta (Figure 4D) (see also Additional file 2). By way of contrast, the GFP fluorescence of the reverse chimera GFP-rEag2-I exhibited rEag1-like punctate localization (Figure 4D) (see also Additional file 3). We also quantified the puncta density of the GFP signal by calculating the number of GFP puncta per neuron. The GFP puncta densities of GFP-rEag1 and GFP-rEag2 were about 153 ± 5 and 17 ± 2, respectively (Figure 4E). This 9-fold difference in GFP puncta density between over-expressed GFP-rEag1 and GFP-rEag2 channels is almost equivalent to the previously measured 8-fold difference in puncta density between endogenous rEag1 and rEag2 proteins (see Figure 2B). The GFP puncta density of the GFP-rEag1-I chimera was dramatically reduced to about 31 ± 7, which is statistically similar to that of GFP-rEag2 (Figure 4E). In contrast, the GFP puncta density of the GFP-rEag2-I chimera was remarkably increased to about 109 ± 6 (Figure 4E); this value, although falling short of that of the GFP-rEag1 density, is more than 6-fold higher than that of the GFP-rEag2 density. When taken together, these findings suggest a potential correlation between the presence of the rEag1 post-CNBHD sequence and the punctate localization of the protein in question in hippocampal neurons.

**Figure 5:**
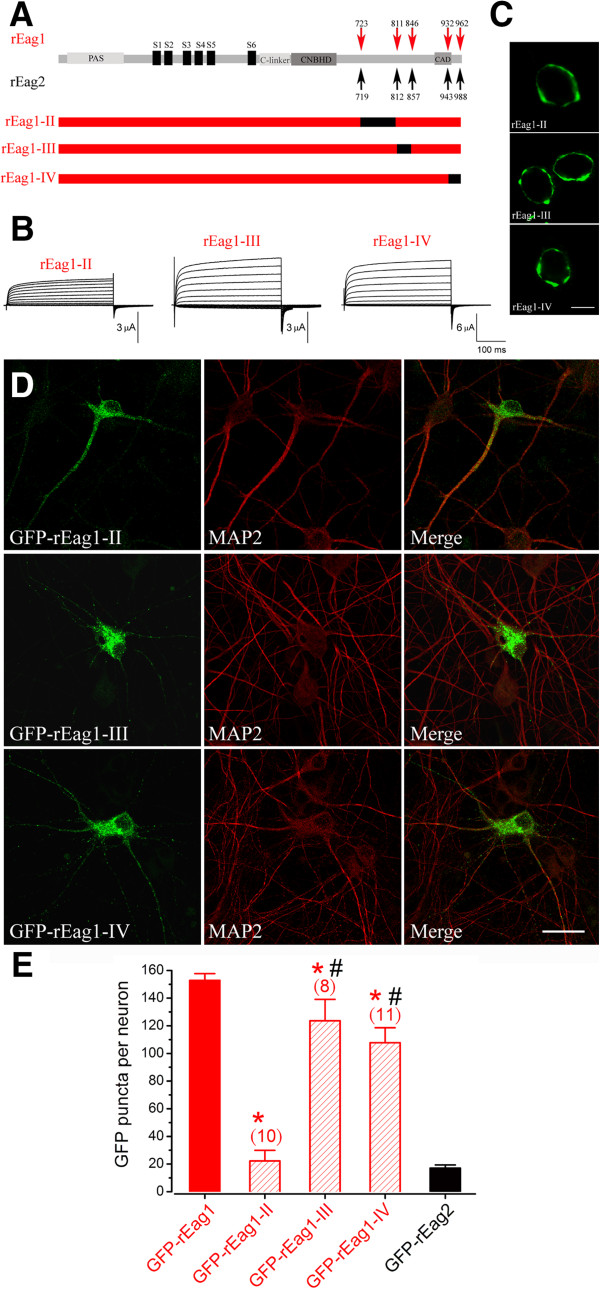
**Characterization of rEag1-II, III, and IV chimeric channels. (A)** Schematic representation of the construction of rEag1-II, rEag1-III, and rEag1-IV chimeras. **(B)** Representative K^+^ currents recorded from *Xenopus* oocytes over-expressing the indicated rEag1 constructs. **(C)** Membrane localization of the GFP-rEag1 chimeric channels in HEK293T cells. Scale bar, 10 μm. **(D)** Expression of the GFP-rEag1 chimeric channels in DIV12 hippocampal neurons. Scale bar, 25 μm. **(E)** Quantification of the number of GFP puncta per neuron for the GFP-rEag1 chimeric channels. Note the presence of rEag2-like GFP puncta density in rEag1-II only. (*: significantly different from GFP-rEag1; *t*-test, p < 0.05)(#: significantly different from GFP-rEag2; *t*-test, p < 0.05)

To further examine whether the post-CNBHD region may contribute to the differential subcellular localization of the two Eag isoforms, we generated three additional rEag1 chimeras, namely rEag1-II, rEag1-III, and rEag1-IV, each of which contains a separate rEag2 post-CNBHD segment with a distinct sequence divergence from that of rEag1 (Figure 5A). In the heterologous expression system, the functional and membrane trafficking properties of all three rEag1 chimeras were similar to those of wild-type rEag1 (Figure 5B-C). We then inspected the subcellular localization of these GFP-tagged chimeras in DIV12 hippocampal neurons (Figure 5D) (see also Additional file 2). A quantitative analysis of the GFP fluorescence results indicated that the GFP puncta densities of GFP-rEag1-II, GFP-rEag1-III, and GFP-rEag1-IV chimeras were about 22 ± 8, 124 ± 15, and 108 ± 11, respectively (Figure 5E). In other words, a rEag2-like pattern with very few GFP puncta was only observed in the GFP-rEag1-II chimera, which is the protein where the proximal post-CNBHD region (rEag1 A723-R807) has been substituted with its rEag2 counterpart (rEag2 L719-G808).

**Figure 6:**
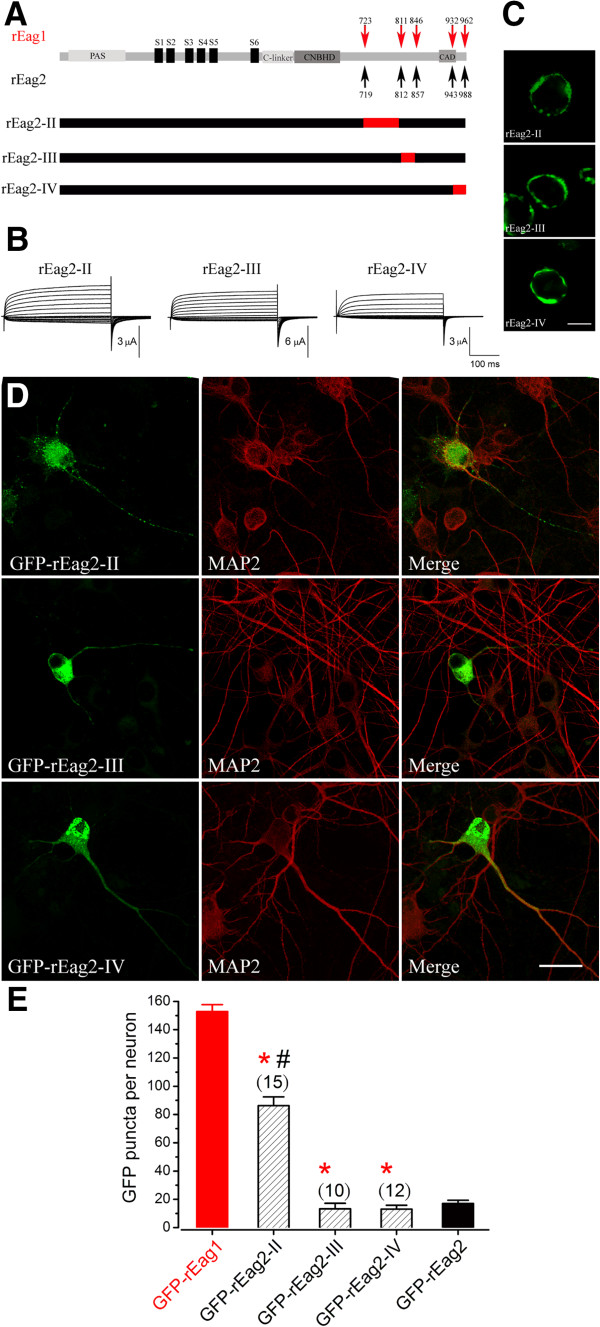
**Characterization of rEag2-II, III, and IV chimeric channels. (A)** Schematic representation of the construction of rEag2-II, rEag2-III, and rEag2-IV chimeras. **(B)** Representative K^+^ currents recorded from *Xenopus* oocytes over-expressing the indicated rEag2 constructs. **(C)** Membrane localization of the GFP-rEag2 chimeric channels in HEK293T cells. Scale bar, 10 μm. **(D)** Expression of the GFP-rEag2 chimeric channels in DIV12 hippocampal neurons. Scale bar, 25 μm. **(E)** Quantification of the number of GFP puncta per neuron for the GFP-rEag2 chimeric channels. Note the presence of rEag1-like GFP puncta density in rEag2-II only. (*: significantly different from GFP-rEag1; *t*-test, p < 0.05)(#: significantly different from GFP-rEag2; *t*-test, p < 0.05)

The above findings imply that the proximal post-CNBHD region of rEag1 is likely to play an essential role in the expression of a punctate localization pattern. To further test this hypothesis, we then constructed three reverse rEag2 chimeras, namely rEag2-II, rEag2-III, and rEag2-IV, each of which harbored a segment of the rEag1 post-CNBHD sequences (Figure 6A). In the heterologous expression system, the three rEag2 chimeras were similar to each other in terms of their functional and membrane trafficking properties (Figure 6B-C). Figure 6D (see also Additional file 3) illustrates representative localization patterns of the GFP-tagged chimeras in hippocampal neurons. The calculated values of GFP puncta densities were about 86 ± 6 (GFP-rEag2-II), 13 ± 4 (GFP-rEag2-III), and 13 ± 3 (GFP-rEag2-IV) (Figure 6E). Therefore, prominent punctate localization was only found when GFP-rEag2-II, the chimera containing the rEag1 segment A723-R807, was present.

**Figure 7:**
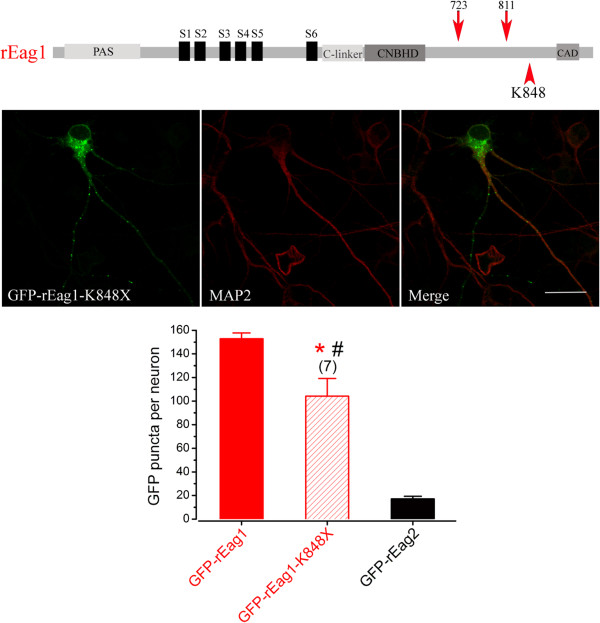
**Expression of GFP-rEag1-K848X channels in hippocampal neurons.** (*Left panel*) GFP-rEag1-K848X was over-expressed in DIV12 hippocampal neurons. Similar to GFP-rEag1 channels, the GFP signal arising from the truncation mutant also displayed the characteristic punctate pattern. Scale bar, 25 μm. (*Right panel*) Quantification of the number of GFP puncta per neuron for rEag1-K848X. (*: significantly different from GFP-rEag1; *t*-test, p < 0.05)(#: significantly different from GFP-rEag2; *t*-test, p < 0.05)

The foregoing observations directly imply that the distal post-CNBHD region, including the carboxyl assembly domain (CAD) (Figure 3C), is not involved in determining the subcellular localization of rEag1. To address this issue, we focused on a previously identified truncation mutant that lacks CAD, K848X, the membrane trafficking and biophysical properties of which are similar to those of wild-type rEag1 [18]. Figure 7 (see also Additional file 4) shows that GFP-rEag1-K848X does indeed display considerable punctate localization in DIV12 hippocampal neurons. The GFP puncta density of GFP-rEag1-K848X (104 ± 15), while less than that of GFP-rEag1, is about 6-fold higher than that of GFP-rEag2, which is consistent with the idea that the distal post-CNBHD region is not required for conferring the punctate localization on rEag1 channels.

### The voltage-dependent gating properties of the chimeric channels

**Figure 8:**
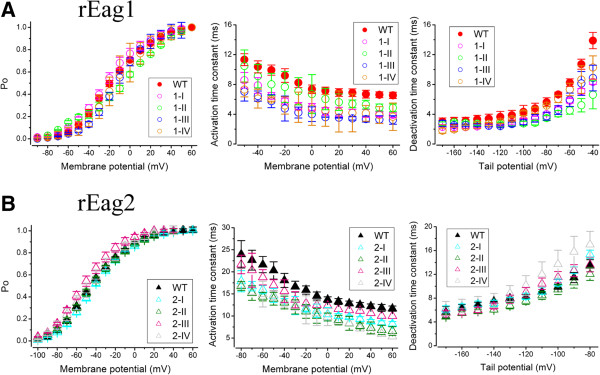
**The biophysical properties of the chimeric Eag channels.** Comparison of the voltage-dependent gating properties of the rEag1 **(A)** or rEag2 **(B)** chimeras with their wild-type (WT) counterparts. Steady-state voltage dependence (activation curve) is illustrated as the fraction of open channels (*P*o) against the corresponding membrane potential. Activation time constants at indicated potentials were obtained from single exponential fits to the late rising phase of Eag K^+^ currents. Deactivation time constants were derived from single exponential fits to the decay phase of Eag K^+^ currents at the indicated tail potential in response to a +40 mV test pulse. All values are presented as mean ± SEM. Data were collected and analyzed as described previously [18].

In addition to divergent subcellular localization patterns, rEag1 and rEag2 channels also have different gating properties including steady-state voltage dependence and activation/deactivation kinetics [4-6]. A similar disparity has also been observed in human Eag1 and Eag2 channels [7,9]. To understand whether sequence divergence in the post-CNBHD region may also contribute to the distinct biophysical properties of the two Eag K^+^ channel isoforms, we went on to analyze the gating property of the chimeras. The left panels in Figure 8, as well as Table 1, demonstrate that the steady-state voltage dependence (P_O_-V curve) properties of the chimeras are similar to those of their wild-type counterparts, indicating that sequence divergence in the post-CNBHD region is not able to account for the ~40-mV discrepancy in voltage activation between rEag1 and rEag2. Furthermore, despite about two-fold difference in the activation kinetics between the two Eag isoforms, exchanging post-CNBHD sequences led to only a small acceleration in the activation kinetics of all chimeras (Figure 8, *middle panels*). Moreover, the introduction of chimeric post-CNBHD sequences did not have any significant effect on the deactivation kinetics of the two Eag isoforms (Figure 8, *right panels*). Taken together, our biophysical findings demonstrate that the sequence differences in the post-CNBHD region are unable to explain the divergence in the voltage-dependent gating properties of rEag1 and rEag2 K^+^ channels.

**Table 1:** Steady-state voltage-dependent activation parameters of the rEag1 and rEag2 chimeric channels

	**V**_**0.5 **_**(mV)**	***k *** **(mV)**	**n**
rEag1-WT	-17.5 ± 0.9	19.9 ± 1.5	16
rEag1-I	-19.2 ± 0.8	17.8 ± 1.2	17
rEag1-II	-8.5 ± 0.7*	24.6 ± 0.7*	11
rEag1-III	-16.1 ± 0.6	14.3 ± 1.5*	15
rEag1-IV	-15.8 ± 1.3	18.3 ± 1.0	12
rEag2-WT	-43.6 ± 1.2	18.3 ± 1.1	18
rEag2-I	-40.0 ± 0.7	17.8 ± 0.9	10
rEag2-II	-42.6 ± 0.5	19.0 ± 0.7	14
rEag2-III	-54.1 ± 1.8*	19.1 ± 1.4	15
rEag2-IV	-44.8 ± 1.4	18.5 ± 1.3	14

To study steady-state voltage-dependent activation of channels, isochronal tail currents were normalized to the maximum amplitude to obtain the fraction of open channels (Po) at the indicated membrane potential. Data points were fit with a Boltzmann equation: Po(V) = 1/{1 + exp[(V_0.5_-V)/*k*]}, where V_0.5_ is the half-maximal voltage for activation, and *k* the slope factor of the Po-V curve. Data are shown as mean ± SEM (*, significantly different from the corresponding wild-type (WT); *t*-test: p < 0.05).

## Discussion

In this report, we began by inspecting the subcellular localization of rEag1 and rEag2 K^+^ channels in young and mature neurons in culture. Our immunofluorescence analysis indicated that, in addition to the dendrosomatic domain, both two Eag isoforms were present in axons of hippocampal neurons. Most importantly, significant punctate distribution was observed in both the dendrosomatic and the axonal compartments for rEag1 channels only. In addition, by over-expressing GFP-tagged constructs, we found that GFP-rEag1, but not GFP-rEag2, displayed significant punctate localization in hippocampal neurons, which is reminiscent of the differential subcellular localization of endogenous rEag1 and rEag2 channels. We further addressed the structural basis underlying the differences in localization patterns and demonstrated that the proximal post-CNBHD region (rEag1 A723-R807) in the C-terminus confers the punctate localization of rEag1 K^+^ channels in hippocampal neurons. To the best of our knowledge, this is the first direct evidence showing that the post-CNBHD region seems to contribute to the subcellular localization of Eag K^+^ channels.

The assessment of protein expression in axons may be hindered by the presence of extensive neurite connections within cultured neurons, as was seen in our previous study of DIV14 hippocampal cultures [12]. In this study we focused on the immunofluorescence characterization of younger neurons where the neurite network is less sophisticated. By closely inspecting DIV3 neurons, wherein one fast-growing neurite becomes the axon and the other slow-growing neurites become the dendrites [19], we observed significant immunofluorescence signal in MAP2-negative, tau-positive neurites for both rEag1 and rEag2 K^+^ channels. Similar results were also found in DIV7 neurons, wherein dendritic spine formation and synaptic connections can be clearly identified [19]. Together with our previous demonstration of their dendrosomatic localization [12], we propose that in hippocampal neurons the two rat Eag isoforms seem to play distinct but essential physiological roles in modulating dendrosomatic excitability, as well as in the propagation of action potential in axons. The foregoing inference is reminiscent of the physiological significance of the prototypic Eag channel that was cloned from *Drosophila*[20]. Electrophysiological recordings from motor neurons in *Drosophila* with mutations in the *eag* gene revealed an increase in spontaneous neuronal firing and presynaptic transmitter release [21], which is consistent with the somatic and axonal localization of *Drosophila* Eag K^+^ channels. Moreover, *Drosophila* with mutations in the *eag* gene were found to be deficient in antennal sensitivity to a subset of odorants [22], which suggests that Eag K^+^ channels may also display dendritic localization in the olfactory receptor neurons of *Drosophila* antennae.

In clear agreement with the conclusion drawn from our previous immunofluorescence studies [12,13], the current study demonstrates that rEag1, but not rEag2, displays significant punctate localization in both the dendrosomatic and the axonal compartments of mature hippocampal neurons. A significant fraction of rEag1 puncta was found to be co-localized with synaptic markers such as synaptophysin [12], densin-180 [12], and PSD-95 (Figure 2A). Furthermore, fractionation analysis revealed that rEag1 was highly enriched in the synaptosomal fraction (Figure 2B). We therefore propose that rEag1 channels are significantly expressed at presynaptic axonal terminals and on postsynaptic dendritic spines, and may play a critical role in controlling neurotransmitter release and postsynaptic signaling.

Specific structural domains have been identified to explain the structure-function mechanisms underlying the divergent voltage-gating processes of different K^+^ channels [23,24]. Similarly, various sequence motifs within different voltage-gated K^+^ channels have been shown to govern their subcellular localization and the targeting of channel proteins to different neuronal compartments [11,15]. Despite the presence of about 70% identity in amino acid sequence between the Eag1 and Eag2 K^+^ channel proteins [4,6], the structural bases of their different voltage-gating properties and subcellular localizations have remained largely elusive. Previous biophysical analysis of a series of different chimeras between human Eag1 and Eag2, for example, revealed that the transmembrane regions alone were not sufficient to explain the differences in their gating kinetics and steady-state voltage-dependence [25,26]. In addition, similar to our results here (Figure 8), non-membrane regions *per se* were found not to determine their gating behaviors [26]. Together these results suggest that the divergent voltage-gating property between the two Eag isoforms may rather arise from interactions among multiple structural domains within the channel protein.

In this study we found that the GFP-tagged rEag1 chimeras (1-I and 1-II) that harbor the proximal post-CNBHD region of rEag2 displayed a dramatic reduction in hippocampal neuron fluorescence puncta (Figures 4E and 5E). Conversely, notable punctate patterns were observed with the GFP-tagged rEag2 chimeras (2-I and 2-II) which contain the proximal post-CNBHD region of rEag1 (Figures 4E and 6E). Finally, the rEag1 truncation mutant K848X that lacks the distal post-CNBHD region still displayed significant punctate localization in hippocampal neurons (Figure 7). Taken these findings as a whole, they strongly support the hypothesis that the punctate localization of rEag1 K^+^ channels is conferred by the proximal post-CNBHD region. However, it remains to be determined whether this region alone is sufficient to determine the pre/post-synaptic localization of rEag1. One alternative is that the synaptic targeting of rEag1 channels may involve interactions between a subset of the proximal post-CNBHD sequences and other protein domains. Furthermore, we cannot rule out the possibility that certain proximal-post-CNBHD-interacting protein(s) may be required for the punctate/synaptic localization of rEag1 protein.

As mentioned in the Results section, for the K848X truncation mutant and the chimeras that do display prominent punctate localization, their GFP puncta densities are about 5-fold to 7-fold higher than that of wild-type rEag2. Nevertheless, the GFP puncta densities of these constructs are still actually lower than that of wild-type rEag1. The reason for this discrepancy is unknown. One possible explanation is that in neurons, the chimeric and mutant constructs are less effective in terms of protein expression, membrane trafficking, and/or puncta formation.

## Conclusions

Immunofluorescence studies reveal that in hippocampal neurons, rEag1 and rEag2 K^+^ channels are present in both the dendrosomatic and the axonal compartments. In addition, rEag1 protein is significantly expressed within synaptic regions and displays a distinct punctate localization pattern. By studying a series of different chimeric constructs between rEag1 and rEag2, we have determined that the proximal post-CNBHD region of the rEag1 protein confers punctate localization of rEag1 K^+^ channels. These findings highlight a new direction for studies in this area and provide important insights that should help the elucidation of the physiological significance of Eag K^+^ channels in the brain.

## Abbreviations

CAD: Carboxyl assembly domain; CNBHD: Cyclic nucleotide-binding homology domain; DIV: Days *in vitro*; Eag: *Ether-à-go-go*; GFP: Green fluorescent protein; MAP2: Microtubule-associated protein 2; PSD: Postsynaptic density; rEag: Rat Eag; SPM: Synaptosomal.

## Competing interests

The authors declare that they have no competing interests.

## Authors’ contributions

GMJ, MMC, and CJJ conceived and designed the experiments. CCC, HML, YJP performed DNA transfection, immunofluorescence, hippocampal culture, and confocal microscopy. CCC, YHW, and JHH carried out electrophysiological experiments and analyses. HML and YHW accomplished mutagenesis and subcloning. YCC conducted fractionation analyses. CJJ wrote the manuscript with input from the co-authors. All authors approved the final version of the manuscript.

## Supplementary Material

Additional file 1 **Additional confocal microscopic images of GFP-rEag1 and GFP-rEag2 channels over-expressed in DIV12 hippocampal neurons.** Two representative cells are shown for each construct. Scale bar, 40 μm. Click here for file

Additional file 2 **Additional confocal microscopic images of the GFP-rEag1 chimeric channels over-expressed in DIV12 hippocampal neurons.** Two representative cells are shown for each construct. Scale bar, 40 μm. Click here for file

Additional file 3 **Additional confocal microscopic images of the GFP-rEag2 chimeric channels over-expressed in DIV12 hippocampal neurons.** Two representative cells are shown for each construct. Scale bar, 40 μm. Click here for file

Additional file 4 **Additional confocal microscopic images of GFP-rEag1-K848X channels over-expressed in DIV12 hippocampal neurons.** Two representative cells are shown. Scale bar, 40 μm. Click here for file
