# Supplementary figures and images for: The punctate localization of rat Eag1 K+ channels is conferred by the proximal post-CNBHD region
Source: BMC Neurosci. 2014 Feb 4;15:23. doi: 10.1186/1471-2202-15-23 (PMC3926332; doi:10.1186/1471-2202-15-23)

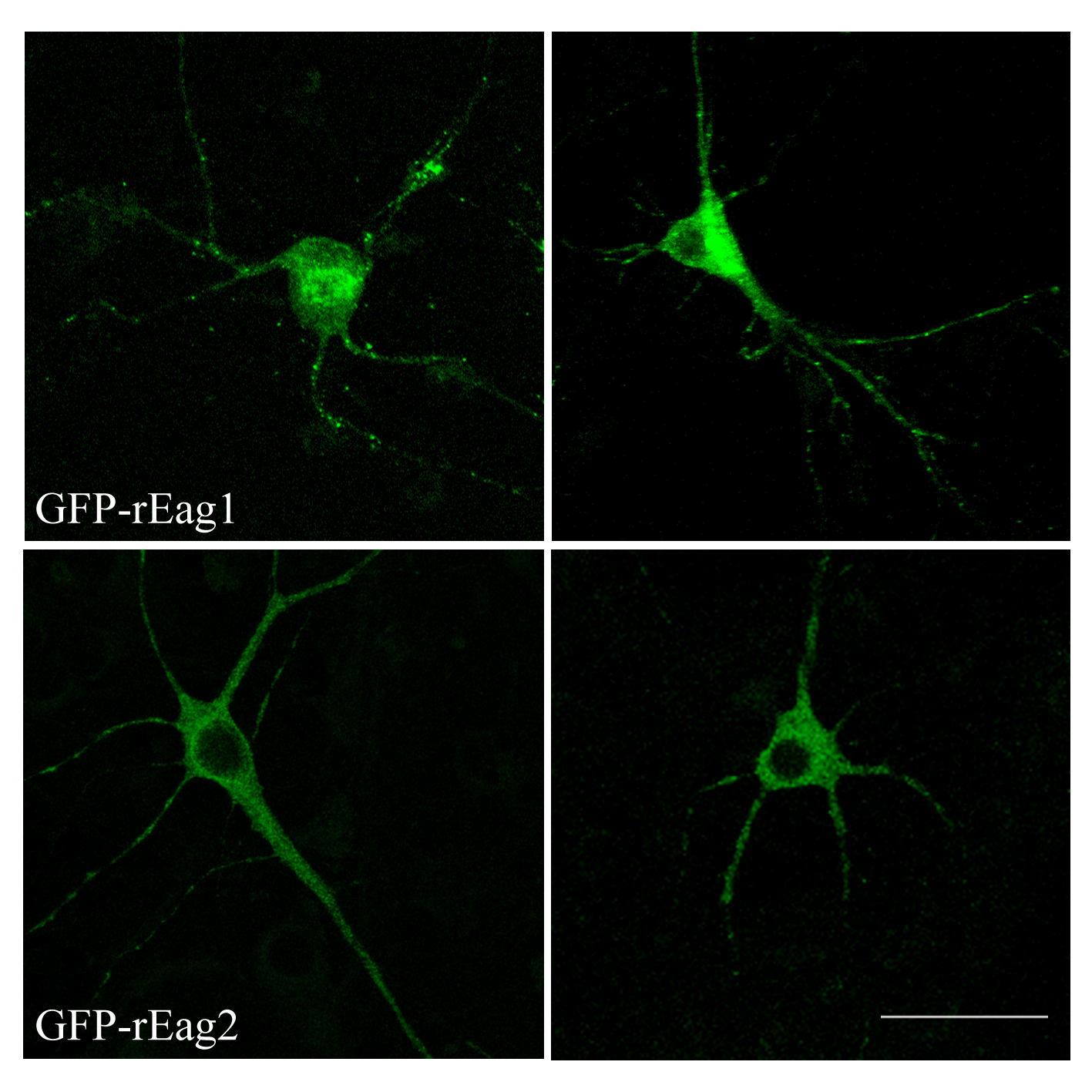

Supplement: Additional file 1 — Additional confocal microscopic images of GFP-rEag1 and GFP-rEag2 channels over-expressed in DIV12 hippocampal neurons. Two representative cells are shown for each construct. Scale bar, 40 μm. [file 1471-2202-15-23-S1.tiff]

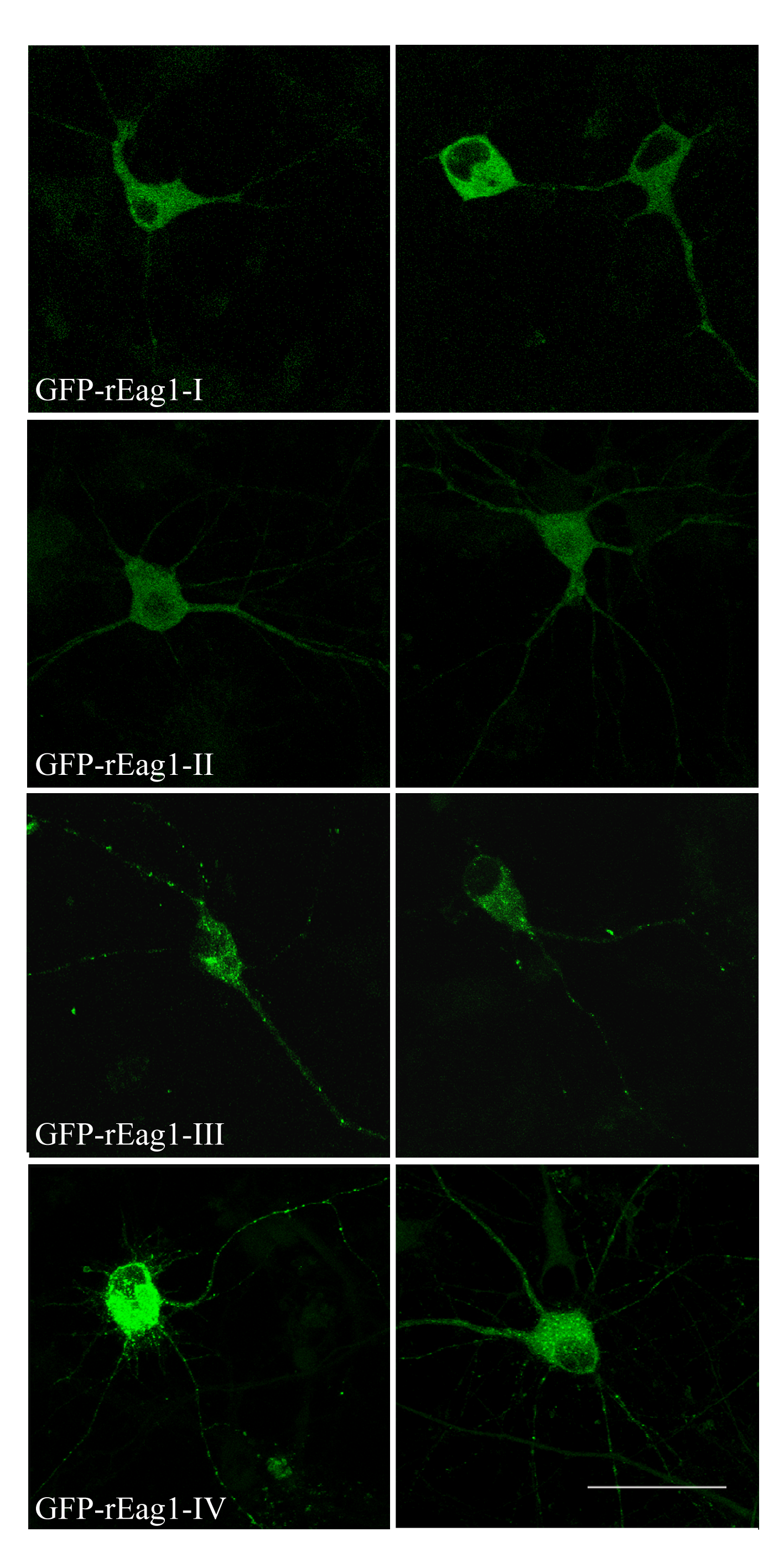

Supplement: Additional file 2 — Additional confocal microscopic images of the GFP-rEag1 chimeric channels over-expressed in DIV12 hippocampal neurons. Two representative cells are shown for each construct. Scale bar, 40 μm. [file 1471-2202-15-23-S2.tiff]

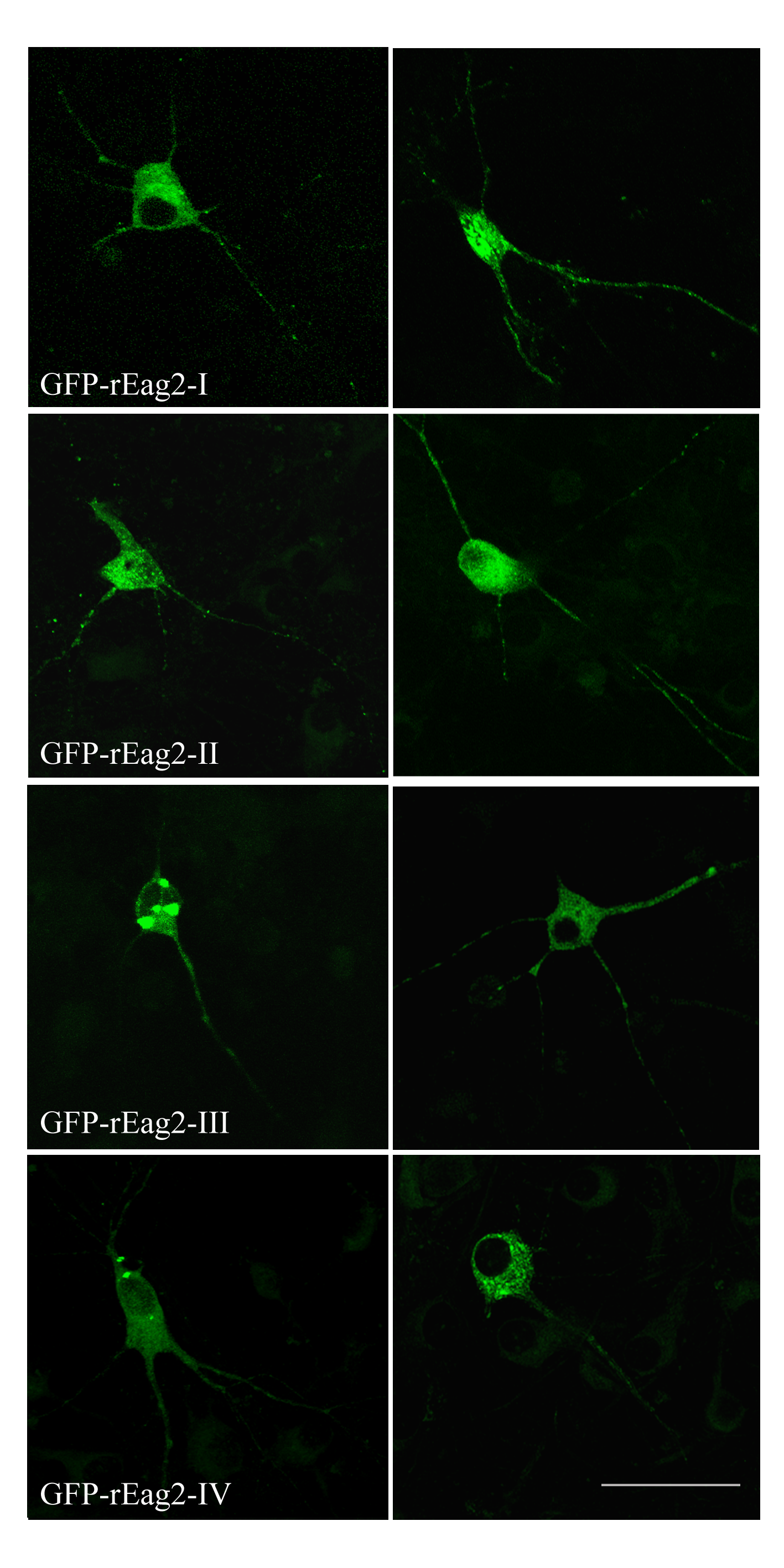

Supplement: Additional file 3 — Additional confocal microscopic images of the GFP-rEag2 chimeric channels over-expressed in DIV12 hippocampal neurons. Two representative cells are shown for each construct. Scale bar, 40 μm. [file 1471-2202-15-23-S3.tiff]

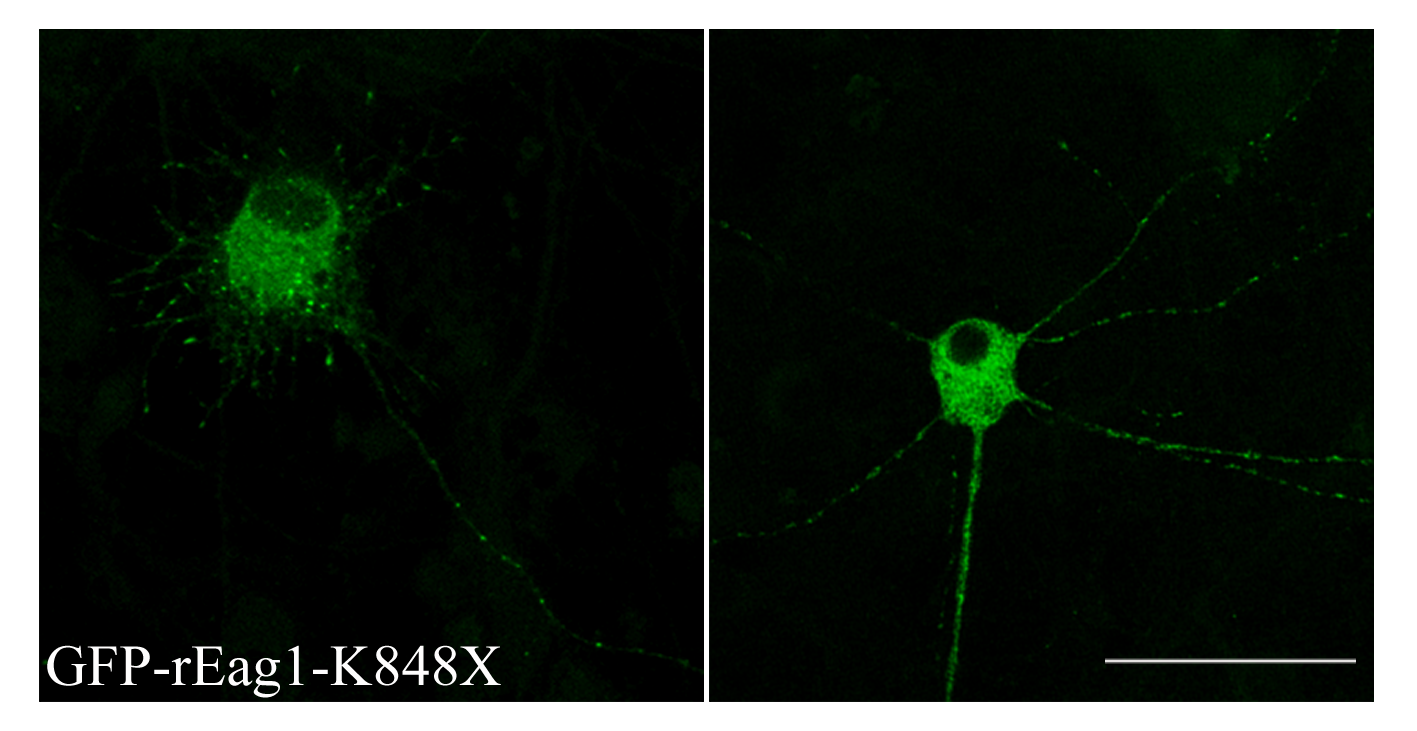

Supplement: Additional file 4 — Additional confocal microscopic images of GFP-rEag1-K848X channels over-expressed in DIV12 hippocampal neurons. Two representative cells are shown. Scale bar, 40 μm. [file 1471-2202-15-23-S4.tiff]
